# Genetic variants in de novo lipogenic pathway genes predict the prognosis of surgically-treated hepatocellular carcinoma

**DOI:** 10.1038/srep09536

**Published:** 2015-03-26

**Authors:** Hequn Jiang, Jingyao Dai, Xiaojun huang, Yibing Chen, Ping Qu, Jibin Li, Cheng Yi, Yefa Yang, Kejing Zhang, Qichao Huang

**Affiliations:** 1 Cancer Center, West China Hospital, Sichuan University, Chengdu, Sichuan 610041, China; 2 Department of Hepatobiliary Surgery, Xijing Hospital, Fourth Military Medical University, Xi'an, 710032, China; 3 State Key Laboratory of Cancer Biology and Experimental Teaching Center of Basic Medicine, Fourth Military Medical University, Xi'an, 710032, China; 4 Department of Interventional Radiology, Eastern Hepatobiliary Surgery Hospital, Second Military Medical University, Shanghai, 200438, China; 5 College of Life Science, Northwest University, Xi'an, 710069, China

## Abstract

Over-expression of de novo lipogenesis (DNL) pathway genes is associated with the prognosis of various types of cancers. However, effects of single nucleotide polymorphisms (SNPs) in these genes on recurrence and death of hepatocellular carcinoma (HCC) patients after surgery are still unknown. A total of 492 primary HCC patients treated with surgery were included in this study. Nine SNPs in 3 genes (ACACA, FASN and ACLY) of DNL pathway were genotyped. Multivariate Cox proportional hazard regression model and Kaplan-Meier curve were used to analyze the association of SNPs with clinical outcomes. Two SNPs in ACACA gene were significantly associated with overall survival of HCC patients. Patients carrying homozygous variant genotype (VV) in rs7211875 had significantly increased risk of death, while patients carrying VV genotype in rs11871275 had significant decreased risk of death, when compared with those carrying homozygous wild-type or heterozygous genotypes. Moreover, patients carrying VV genotype in rs11871275 had decreased recurrence risk, while patients carrying variant genotype in rs4485435 of FASN gene had increased recurrence risk. Further cumulative effect analysis showed significant dose-dependent effects of unfavorable SNPs on both death and recurrence. SNPs in DNL genes may serve as independent prognostic markers for HCC patients after surgery.

Hepatocellular carcinoma (HCC) is the fifth most common solid tumor and the fourth leading cause of cancer-related death worldwide, especially in East Asia. Even with aggressive treatment, HCC usually has a poor prognosis, with a 5-year survival rate as low as 25%–39% after standard treatment^1^. Traditional clinicopathological parameters such as imaging parameters, serum alpha fetoprotein level and tumor stage only offer limited efficacy for prognosis prediction and fail to effectively guide the individualized therapeutics for HCC^2^. Therefore, it is extremely urgent to develop novel biomarkers to discriminate patient groups with different clinical outcomes and improve the treatment for HCC patients.

One of the important features of cancer cells is the increased de novo lipogenesis (DNL) for the purpose to meet the excessive bioenergetic and structural demands of proliferation, irrespective of the extracellular lipid level^3^. The newly synthesized lipids have also been demonstrated to mediate cell migration, signal transduction, intracellular trafficking by constructing membrane rafts^4^,^5^,^6^. Thus, the enhanced de novo lipogenesis (DNL) plays important roles in tumor cell survival^7^,^8^,^9^,^10^. At the molecular level, enhanced DNL is reflected by the coordinately increased activity or expression of lipogenic enzymes in neoplastic cells^3^. There are three key rate-limiting enzymes involved in DNL pathway, *i.e.* ATP citrate lyase (ACLY), acetyl-CoA carboxylase alpha (ACACA) and fatty-acid synthase (FASN). ACLY converts citrate into cytosolic acetyl-CoA, which is transformed into malonyl-CoA by the catalysis of ACACA. The malonyl-CoA product is further converted by FASN to long-chain fatty acids. Numerous studies have demonstrated that DNL enzymes are overexpressed in various human cancer types, including liver cancer^11^,^12^. Furthermore, the overexpression of FASN or ACACA in tumor tissues has been associated with poor clinical outcomes of prostate, breast, nasopharyngeal, colon, ovarian and lung cancer patients^5^,^13^,^14^,^15^,^16^,^17^,^18^. ACLY is upregulated in several types of cancer cells and this enzyme also plays an important role in cancer cell progression^19^. Therefore, molecules in DNL pathway may have potentials to serve as prognostic markers for cancer patients.

Single nucleotide polymorphism (SNP), a common typical genomic variation, can be used as a stable biomarker of genetic background to profile and predict some useful characteristics about cancer, including the susceptibility, development of cancer, and patients' prognosis and response to treatment. Also, genetic variations may be important cause factors that influence the expression or catalytic activity of DNL enzymes, and subsequently modulate their biological functions^20^. Quite a few studies have investigated the relationship between single nucleotide polymorphisms (SNPs) of DNL genes and development or prognosis of several tumors such as uterine leiomyomata, breast and prostate cancers^21^,^22^,^23^,^24^. However, no studies have focused on the association between polymorphisms of DNL genes and HCC prognosis until now. Therefore, we assessed the effects of nine SNPs in the three key genes of DNL pathway on the prognosis of HCC patients in a cohort of 492 Chinese subjects.

## Methods

### Study Population

Between January 2009 and January 2012, a total of 518 HCC patients were enrolled at Xijing Hospital affiliated with Fourth Military Medical University in Xi'an and the Eastern Hepatobiliary Surgery Hospital affiliated with Secondary Military Medical University in Shanghai, China. Patients with histologically confirmed primary HCC were eligible for the enrollment. There were no recruitment restrictions on age, gender and tumor stage. All HCC diagnosis was based on the National Comprehensive Cancer Network (NCCN) clinical practice guidelines in oncology. In the present study, we excluded 26 patients, including 3 patients with incomplete clinical data and 23 patients who were dead within 1 month post-surgery. Finally, a total of 492 eligible HCC patients were recruited, with complete and validated demographic, clinical, and follow-up data. All patients received surgery within 2 months after diagnosis. The primary tumor was completely resected for all patients, and was confirmed by pathological review of the tumors after resection. No patient received anticancer treatment before surgery. The study was approved by the Ethical Committees of Fourth Military Medical University and Secondary Military Medical University. Written consent was obtained from each patient. All experiments involving human subjects and tissues were performed in accordance with guidelines approved by Fourth Military Medical University and Secondary Military Medical University.

### Epidemiologic and clinical data collection

Demographic data were collected through in-person interviews at the time of initial visit or follow-up in the clinics, medical chart review, or consultation with the treating physicians by trained clinical research specialists. For data acquired from multiple sources, the research staff compared and validated the consistency of these data. The patients and/or family members were further contacted for verification if there were discrepancies. Disease stage was assessed using the seventh American Joint Committee on Cancer (AJCC) and Barcelona Clinic Liver Cancer (BCLC) staging systems. The follow-up information was updated at 6-month intervals through onsite interviews, direct calling, or medical chart review. The latest follow-up in this study was carried out in January 2013.

### SNP selection and genotyping

Genomic DNA was obtained for each participant as described previously^25^. Three DNL pathway genes (*ACACA*, *FASN* and *ACLY*) were included as candidate genes in this study to estimate the effect of their genetic polymorphisms on the survival in HCC patients. SNPs in these genes were selected using a set of web-based SNP selection tools (http://snpinfo.niehs.nih.gov/snpinfo/snpfunc.htm) as previously described^26^. Briefly, the strategy and criteria for the selection of SNPs were as follows: a) located in target genes or their 2000 bp flanking regions; b) potentially functional SNPs had priority; c) had minor allele frequency (MAF) >5% in the Asian population (CHB). If there were multiple potentially functional SNPs within the same block (defined by the linkage coefficient *r*^*2*^ > 0.80), only 1 SNP was included. Functional SNPs included missense SNPs in exons, and SNPs in microRNA binding sites of 3′ untranslated region, transcription factor binding site of the 5′ flanking region and splice sites.

Through the selection process, 9 SNPs were selected for prognosis analysis in this study, including 3 SNPs in transcription factor binding site sequences (rs11871275, rs9912300, and rs11653012), 2 missense SNPs (rs7211875 and rs2304497), 2 synonymous SNPs (rs1140616 and rs4485435); 1 splice site SNP (rs1714987), and 1 SNP with unknown function (rs4246444 PMID: 21646813) (Table S1). Genotyping was performed with iPLEX (Sequenom, San Diego, CA) matrix-assisted laser desorption/ionization time-of-flight mass spectrometry technology. Laboratory personnel conducting genotyping were blinded to patient information. Strict quality control measures were implemented during genotyping with more than 99% concordance in samples that were randomly selected to be genotyped in duplicate.

### Immunohistochemical staining of ACACA and FASN

Formalin-fixed, paraffin-embedded HCC tissues from 64 patients (including 57 males and 7 females) were collected. The median age of these patients were 54 years (range 18–78). There were 52 cases were diagnosed with TNM stage I or II diseases, while 20 with well- or moderately-differentiated tumors. H&E slides from these patients were viewed under a light microscope by a pathologist and 4-μm-thick tissue sections were cut from corresponding blocks containing representative tumor regions. After deparaffinization with dimethylbenzene, the tissue sections were rehydrated through 100%, 95%, 90%, 85% and 75% ethanol. After three washes in phosphate-buffered saline (PBS), every five minutes, the slides were boiled in antigen retrieval buffer containing 0.01 mol/L citrate antigen retrieval (pH = 6.0) in the pressure cooker and then rinsed in peroxidase quenching solution (Invitrogen) to block endogenous peroxidase. The sections were then incubated with a rabbit anti-human ACACA antibody (1:200, Abcam) or rabbit anti-human FASN antibody (1:200, Abcam) at 4°C overnight and then with an Broad Spectrum Second Antibody (Invitrogen) at 37°C for 20 min. After three washes, the visualization signal was developed with Invitrogen Histostain Plus kit. The intensity and extent of immunostaining were evaluated for all samples under double-blinded conditions. In brief, the percentage of positive staining was scored as 0 (0–9%), 1 (10%–25%), 2 (26%–50%), 3 (51%–75%) or 4 (76%–100%), and the intensity as 0 (no staining), 1 (weak staining), 2 (moderate staining) or 3 (dark staining). The total score was calculated as the product of intensity and extent, ranging from 0 to 12.

### Statistical analysis

All statistical analyses were performed using the SPSS Statistics 19.0 software (IBM). The 3 genetic models (additive, dominant, and recessive) were applied to assess the association of single SNPs with clinical outcome of HCC patients. The best-fitting model was defined as that with the smallest *P* value. Only the result predicted by the best model was reported and considered in the subsequent analysis. Hazard ratio (HR) was estimated in a multivariate Cox proportional hazard model, adjusting for age, gender, TNM stage, tumor differentiation, and treatment regimen. The cumulative effect of unfavorable genotypes on OS or RFS was estimated in Cox model^27^. Adjusted Kaplan–Meier curve and log-rank test were used to assess the differences in overall and recurrence-free survival (OS and RFS respectively) between patients with different genotypes^28^. All statistical analyses were two-sided, and *P* < 0.05 was considered to be of statistical significance.

## Results

### Clinical characteristics and their effects on prognosis of HCC patients after surgery

A total of 492 HCC patients were included in this study, with a median age of 53 years (ranging from 18 to 79 years). More than 90% of patients (*n* = 446) were positive for serum HBsAg. The numbers of patients in different TNM stages were as follows: 394 for stage I and II (80.1%), 98 for stage III and IV (19.9%). The majority (*n* = 344, 70.1%) of patients had poor differentiated or undifferentiated tumors, while 57.5% (*n* = 283) had a tumor size larger than 5.0 cm, and 19.5% (*n* = 96) had multiple lesions. More than 70% (*n* = 362) of patients were at BCLC stage A and 37.6% (*n* = 185) received adjuvant transcatheter arterial chemoembolization treatment (TACE).

During the median follow-up of 21.8 months (ranging from 1.6 to 48.3 months), 188 (38.2%) patients died of HCC and 309 developed recurrence. Multivariate Cox regression analysis showed that serum alpha fetoprotein (AFP) level, tumor differentiation, TNM stage, BCLC stage and PVTT had significant influence on overall survival (OS) and recurrence-free survival (RFS) for HCC patients (*P* < 0.01 for all). Adjuvant TACE therapy has been shown to reduce risks of both death (HR, 0.62; 95% CI, 0.45–0.85; *P* < 0.01) and recurrence (HR, 0.56; 95% CI 0.43–0.71; *P* < 0.01) (Table 1).

### Association of single SNP with clinical outcomes of HCC patients

**Figure 1:**
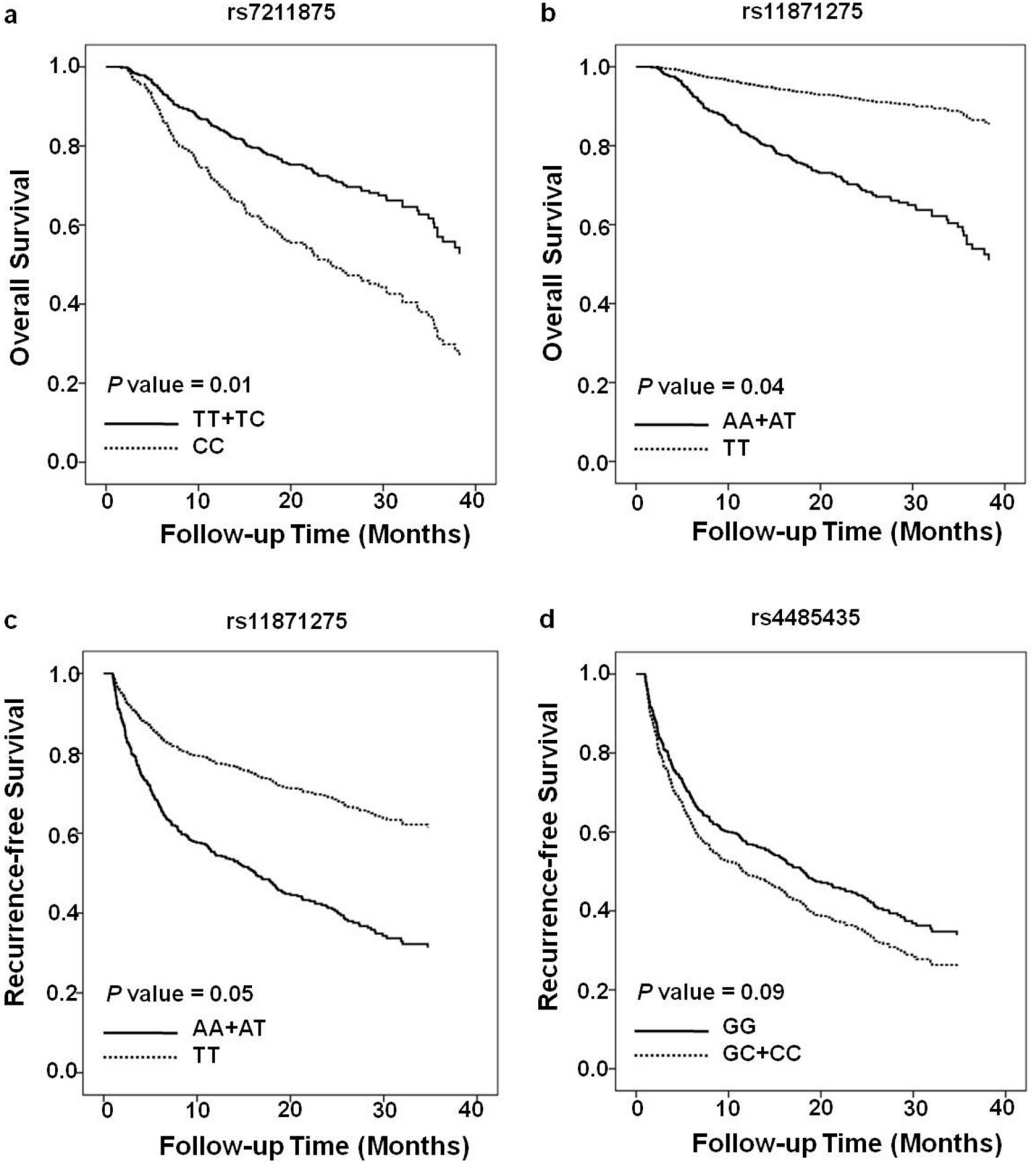
Survival curves of OS (A and B) and RFS (C and D) of HCC patients stratified by SNPs. Homozygous wild-type genotype, heterozygous genotype and homozygous variant genotype for rs7211875 was TT, TC, CC, respectively; for rs11871275 was AA, AT, TT, respectively; for rs4485435 was GG, GC, CC, respectively.

We assessed the effects of 9 SNPs in three DNL pathway genes (*ACACA, FASN* and *ACLY*) on the death and recurrence in HCC patients using the multivariate cox regression model (Table 2). After adjusting for gender, age, HBsAg status, AFP level, TNM stage and differentiation, our data showed that SNPs rs7211875 and rs11871275 in *ACACA* gene were significantly associated with the death risk of HCC patients under recessive model (for rs7211875, HR = 2.13, 95% CI = 1.17–3.88; *P* = 0.01; for rs11871275, HR = 0.22, 95% CI = 0.06–0.91; *P* = 0.04). Adjusted survival curve showed similar results, indicating that HCC patients carrying the homozygous variant (CC) genotype in rs7211875 had significantly worse OS than those carrying the other genotypes (*P* = 0.01, Figure 1A), whereas patients with homozygous variant (TT) genotype in rs11871275 had better OS than those with other genotypes (*P* = 0.04, Figure 1B). Moreover, our data showed that TT genotype in rs11871275 of ACACA gene reduced the recurrence risk of HCC when compared to patients with AA and AT genotypes (HR = 0.41, 95% CI = 0.17–1.00; *P* = 0.05), whereas variant genotype (GC/CC) in rs4485435 of *FASN* gene was associated with increased recurrence risk, when compared to patients with GG genotype (HR = 1.32; 95% CI = 1.02–1.72, *P* = 0.03). Kaplan-Meier analysis confirmed these findings, showing that HCC patients carrying the TT genotype in rs11871275 had better OS than those carrying the other genotypes (*P* = 0.05, Figure 1C), while patients with GC/CC genotypes in rs4485435 trended to show worse OS than did those with GG genotype (*P* = 0.09, Figure 1D).

### Cumulative effects of unfavorable genotypes on the prognosis of HCC patients

**Figure 2:**
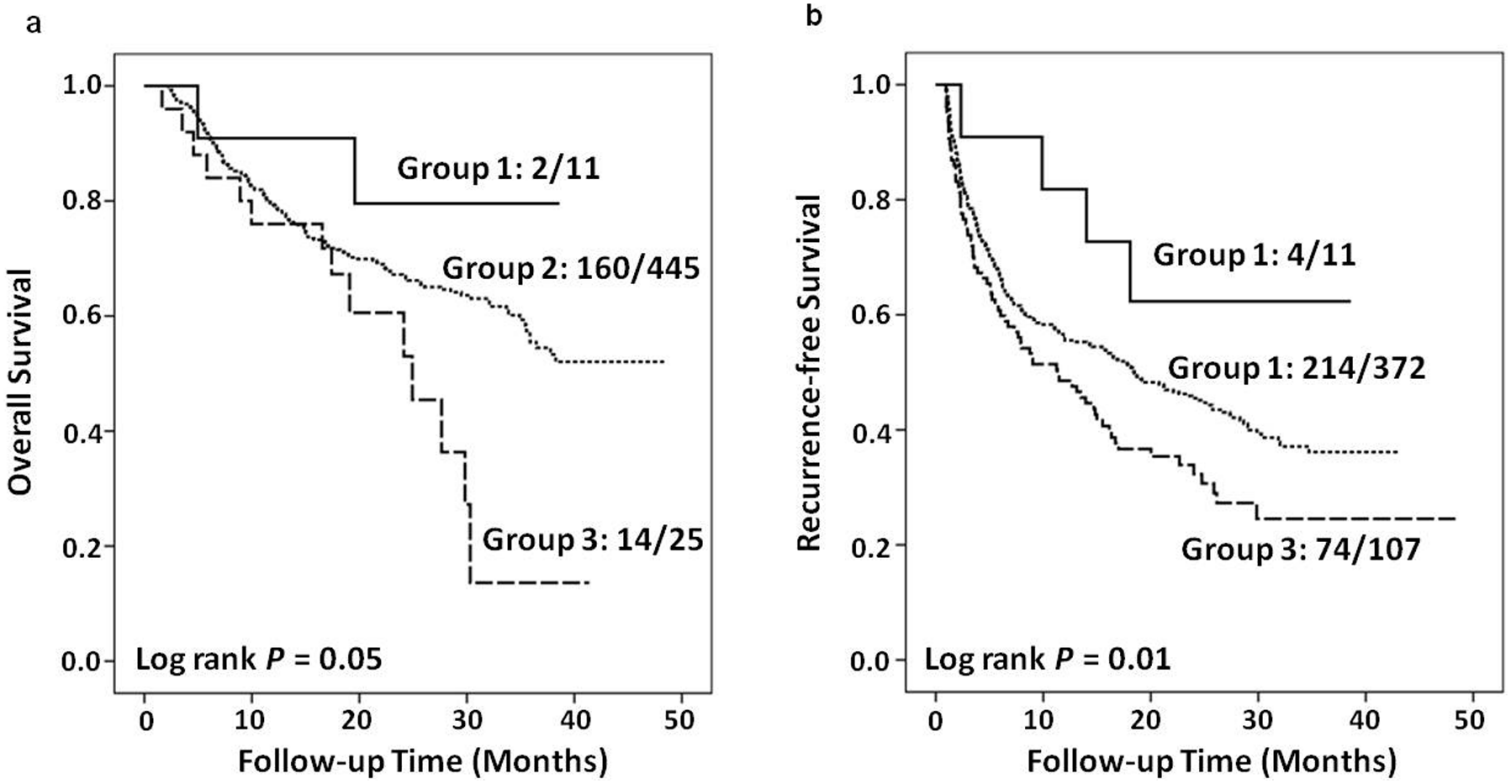
The cumulative effects of unfavorable genotypes in *ACACA* and *FASN* genes on overall (A) and recurrence-free (B) survival of HCC patients analyzed by Kaplan-Meier curves. Group1, patients with 0 unfavorable genotype; Group2, patients with 1 unfavorable genotype; Group3, patients with 2 unfavorable genotypes.

In order to assess the cumulative effects of genetic variants on HCC patients' outcomes, we did a joint analysis by including the three unfavorable genotypes determined by the single SNP analysis. Briefly, homozygous variant genotype (CC) for rs7211875, homozygous wild-type and heterozygous genotype (AA+AT) for rs11871275 and homozygous wild-type and heterozygous genotype (GC+CC) for rs4485435 were defined as the unfavorable genotypes. When using group 1 (with 0 unfavorable genotype) as reference, HCC patients in group 3 (with 2 unfavorable genotypes) had a 6.3-fold increased risk of death (95% CI, 1.41–28.16; *P* < 0.01) (Table 3). Furthermore, when compared with group 1 with 0 unfavorable genotype, increased recurrence risk was observed in group 2 with 1 unfavorable genotype (HR, 2.72; 95% CI, 1.00–7.34; *P* = 0.05) and group 3 with 2 unfavorable genotypes (HR, 3.43; 95% CI, 1.25–9.43; *P* = 0.02) (Table 4). The cumulative death or recurrence risks of unfavorable genotypes exhibited significant dose-dependent manners with both *P* for trend <0.01. Similarly, Kaplan-Meier curves showed significant different OS and RFS among HCC patient groups stratified by number of unfavorable genotypes (*P* = 0.05 and *P* = 0.01, respectively, Figure 2).

### Relationship between SNP genotypes and the expression of ACACA and FASN

We have examined the expression of ACACA and FASN in 64 HCC tissue samples using immunohistochemistry. As showed in supplementary figure 1, patients carrying AT or AA genotype of rs11871275 exhibited significantly higher expression of ACACA when compared with those carrying TT genotype, while the genotypes of SNP rs7211875 and rs4485435 did not show significant association with the expression of FASN. Due to the limited sample size, our results were very preliminary and need to be validated in larger cohort.

## Discussion

In the present study, we evaluated the effects of 9 SNPs in three DNL pathway genes (*ACACA*, *FASN* and *ACLY*) on the prognosis of Chinese HCC patients with surgical resection. Our data demonstrated that SNPs rs7211875 and rs11871275 in the *ACACA* gene were significantly associated with the OS of HCC patients, whereas rs11871275 in *ACACA* gene and rs4485435 in *FASN* gene were significantly associated with the RFS of patients. Furthermore, we found an accumulative risk of both death and recurrence in HCC patients with increasing number of unfavorable genotypes. To the best of our knowledge, this is the first study to report significant effects of genetic variants in DNL pathway genes on the clinical outcomes of HCC patients.

It has been well recognized that the up-regulation of the fatty acid biosynthetic activity starts at a relatively early stage in various types of tumors^29^,^30^. Cancer cells seem to be highly dependent on DNL for their proliferation and survival^29^. Recently, quite a few studies have demonstrated that SNPs in DNL pathway genes are associated with the risk and prognosis of several tumors^21^,^22^,^23^,^24^. For example, it has been reported that SNPs in the *FASN* gene are associated with the risk of uterine leiomyomata^21^, while SNPs in the *ACACA* gene are associated with the risk of breast cancer^22^,^23^. Nguyen PL *et al.* have demonstrated that SNPs in *FASN* gene are associated with the risk and prognosis of prostate cancer^24^. However, no studies have focused on the association between polymorphisms of DNL pathway genes and HCC prognosis until now.

In the present study, we found that two SNPs (rs7211875 and rs11871275) in the *ACACA* gene were significantly associated with the risk of death of HCC patients, while SNP rs11871275 in *ACACA* and rs4485435 in *FASN* gene were associated with the RFS of HCC patients. Our findings are supported by previous studies. Calvisi DF *et al.* has reported that induction of DNL pathway, an important effector pathway of the oncogenic AKT/mTORC1 axis, has pathogenic and prognostic significance for HCC^31^. Moreover, elevated FASN expression is associated with the progression of HCC and poor prognosis of patients^32^. Compared with FASN, little is known about the biological functions and clinical significance of ACACA in HCC. However, marked elevation of ACACA expression and activity has been reported in breast cancer cells^33^,^34^. In addition, ACACA expression is essential to promote breast cancer cell survival^10^. Since rate-limiting catalysis of ACACA in fatty acid elongation in DNL pathway, it is rational to hypothesis that ACACA may also play an important role in the development of HCC.

The biological function of the three SNPs and the molecular mechanisms through which they affect gene functions and clinical outcome of HCC remain to be elucidated. Silicon analysis (including more than 80 bp upstream and downstream of alternative site) with University of California Santa Cruz genome browser (http://genome.ucsc.edu/) indicated that rs4485435 in the exon 21 of *FASN* may be involved in the alternative splicing of *FASN* mRNA. Transcription factor binding sites (TFBS) analysis demonstrated that three SNPs, rs7211875, rs11871275 and rs4485435 are close to one or two TFBS sites, indicating their potential impact on the transcription of DNL genes. MultiZ's alignment^35^ estimated that none of the three SNPs are located in the highly conserved regions of the genome. Our preliminary immunohistochemical analysis showed that that AT+AA genotypes of rs11871275 were significantly associated with higher expression level of ACACA when compared to TT genotype, which is in line with notion that elevated ACACA expression promotes the proliferation of cancer cells. However, no significant association of other SNPs with the expression level of either ACACA or FASN, suggesting that these SNPs may affect the biological activity but not the expression of these proteins. Therefore, these findings need to be validated in larger patient cohort and their biological functions need to be further investigated. Moreover, we did not observe any significant association between 2 functional SNPs in ACLY gene and the prognosis of HCC patients. Possible explanation for our results is that these two SNPs may not or just very weakly affect the expression and functions of ALCY in HCC, which need further investigation in future study.

Various cellular pathways and a complex molecular network may play key roles in the development of HCC. Given the limited power of single SNP in predicting progression of a given disease, we applied a pathway-based approach in our study to evaluate the biological interplay between genes. Our data provide insights into the way by which multiple genes contribute to the pathogenesis of HCC. Furthermore, in this study, we also did a joint analysis by including the three unfavorable genotypes and found a significant dose-dependent effect of the unfavorable genotypes in DNL gene on the OS and RFS. Our results indicate that more powerful prognostic or predictive value can be obtained based on the combination of single SNPs. Therefore, the combination of patients' clinicopathological parameters and genetic variants status of DNL pathway will promote the accuracy and sensitivity of prediction and help improve the clinical management for HCC patients.

Our study has several strengths. First, our pathway-based approach is a logical extension of the candidate gene method, avoiding the requirement of a much larger sample size for a genome-wide association study with free-hypothesis method. Second, the patients are recruited from two large liver disease clinics in China, which can help to minimize the potential selection bias. Our study also has limitations. First, our sample size may not be large enough to detect minimal associations and interactions. Second, we recognize that our follow-up time is relatively short (median follow-up time, 21.8 months; range, 1.6–48.3 months). However, the disadvantage may not affect our results too much because our population has already presented with a considerable percentage of deaths and recurrence.

## Conclusions

Overall, as the first study observing the impact of DNL gene variants on HCC prognosis, our data strongly suggest that SNPs in ACACA and FASN genes may serve as independent prognostic markers for OS and RFS prediction in HCC patients after surgery treatment. These findings warrant validation in other patient populations and further studies on the mechanism underlying the effects of these SNPs on prognosis in HCC.

## Author Contributions

Q.H. and K.Z. conceived and designed the experiments. H.J. and J.D. performed the experiments. P.Q., J.L. and Y. C. analyzed the data. Y.Y., J.H. and C.Y. contributed sample collection. All authors reviewed the manuscript.

## Supplementary Material

Supplementary Information supplementary figure 1 and table 1

## Figures and Tables

**Table 1:** Selected characteristics of HCC patients and prognosis analysis

Variables	No. (%)	OS			RFS		
		Death (%) *n* = 188	HR^a^ (95% CI)	*P* value	Recurrence (%) *n* = 309	HR^a^ (95% CI)	*P* value
Gender							
Female	67 (13.6)	20 (11.2)	Ref.		34 (11.5)	Ref.	
Male	425 (86.4)	159 (88.8)	1.68 (1.04–2.70)	**0.03**	261 (88.5)	1.52 (1.05–2.18)	**0.02**
Age, years							
<53	253 (51.4)	93 (52.0)	Ref.		160 (54.2)	Ref.	
≥53	239 (48.6)	86 (48.0)	1.20 (0.88–1.62)	0.24	135 (45.8)	1.02 (0.81–1.30)	0.85
HBsAg							
Negative	46 (9.3)	16 (8.9)	Ref.		27 (9.2)	Ref.	
Positive	446 (90.7)	163 (91.1)	0.99 (0.58–1.69)	0.96	268 (90.8)	1.07 (0.71–1.61)	0.75
Serum AFP, ng/ml							
<200	264 (53.7)	68 (38.0)	Ref.		138 (46.8)	Ref.	
≥200	228 (46.3)	111 (62.0)	1.98 (1.44–2.72)	**<0.01**	157 (53.2)	1.39 (1.09–1.78)	**<0.01**
Tumor size (%), cm							
<5	209 (42.5)	53 (29.6)	Ref.		106 (35.9)	Ref.	
≥5	283 (57.5)	126 (70.4)	1.57 (1.13–2.20)	**<0.01**	189 (64.1)	1.31 (1.02–1.69)	**0.03**
Tumor number							
Single	396 (80.5)	127 (70.9)	Ref.		220 (74.6)	Ref.	
Multiple	96 (19.5)	52 (29.1)	1.10 (0.73–1.65)	0.65	75 (25.4)	1.32 (0.95–1.84)	0.10
Differentiation grade							
I+II	147 (29.9)	28 (15.6)	Ref.		65 (22.0)	Ref.	
III+IV	344 (70.1)	151 (84.4)	2.29 (1.51–3.49)	**<0.01**	230 (78.0)	1.75 (1.31–2.33)	**<0.01**
TNM stage							
I+II	394 (80.1)	114 (63.7)	Ref.		215 (72.9)	Ref.	
III+IV	98 (19.9)	65 (26.3)	2.85 (2.07–3.91)	**<0.01**	80 (27.1)	2.06 (1.57–2.70)	**<0.01**
BCLC stage							
A	362 (73.6)	101 (56.4)	Ref.		190 (64.4)	Ref.	
B+C	130 (26.4)	78 (43.6)	1.63 (1.16–2.28)	**<0.01**	47 (15.9)	1.62 (1.23–2.13)	**<0.01**
PVTT							
Negetive	439 (89.2)	139 (77.7)	Ref.		247 (83.7)	Ref.	
Positve	53 (10.8)	40 (22.3)	2.36 (1.48–3.78)	**<0.01**	48 (16.3)	2.07 (1.37–3.14)	**<0.01**
Treatment							
Surgery	307 (62.4)	114 (63.7)	Ref.		193 (65.4)	Ref.	
Surgery+TACE	185 (37.6)	65 (36.3)	0.62 (0.45–0.85)	**<0.01**	102 (34.6)	0.56 (0.43–0.71)	**<0.01**

Abbreviations: HBsAg, Hepatitis B virus surface antigen; AFP, alpha fetoprotein; BCLC, Barcelona Clinic Liver Cancer; PVTT, portal vein tumor thrombosis; TACE, transcatheter arterial chemoembolization treatment; RFS, recurrence-free survival; CI, confidence interval; HR, hazard ratio; Ref., reference; OS, overall survival.

^a^Adjusted by age, gender, HBsAg, differentiation, tumor stage, serum AFP and treatment where appropriate.

**Table 2:** Association of single SNPs in DNL pathway related genes with clinical outcome of HCC patients

Gene	SNP	Best fitting Model	OS		RFS	
			HR^a^ (95% CI)	*P* value	HR^a^ (95% CI)	*P* value
*ACACA*	rs1714987	Additive	1.17 (0.94–1.45)	0.16	1.16 (0.99–1.37)	0.07
	rs7211875	Recessive	**2.13 (1.17–3.88)**	**0.01**	1.18 (0.71–1.96)	0.53
	rs11871275	Recessive	**0.22 (0.06–0.91)**	**0.04**	**0.41 (0.17–1.00)**	**0.05**
*ACLY*	rs2304497	Additive	0.84 (0.52–1.37)	0.49	0.76 (0.52–1.12)	0.16
	rs9912300	Dominant	0.78 (0.56–1.08)	0.13	0.83 (0.64–1.07)	0.14
*FASN*	rs4246444	Dominant	0.89 (0.63–1.28)	0.54	0.97 (0.74–1.28)	0.85
	rs1140616	Dominant	0.86 (0.64–1.17)	0.34	0.81 (0.64–1.02)	0.08
	rs4485435	Additive	1.07 (0.77–1.49)	0.70	**1.32 (1.02–1.72)**	**0.03**
	rs11653012	Additive	1.12 (0.79–1.59)	0.51	1.15 (0.89–1.50)	0.29

Abbreviations: CI, confidence interval; HR, hazard ratio; Ref., reference; OS, overall survival; RFS, recurrence-free survival.

^a^Adjusted by gender, age, HBsAg, AFP level, TNM stage, Differentiation, treatment.

**Table 3:** Cumulative Effect of Unfavorable Genotypes on Overall Survival of HCC Patients

Group (Number of Unfavorable Genotype)^a^	Death/Total	HR(95% CI)^b^	*P*-value
Group 1(0)	2/11	Ref.	
Group 2(1)	160/445	2.62(0.65–10.64)	0.18
Group 3(2)	14/25	**6.30(1.41–28.16)**	**<0.01**
*P* for trend			**<0.01**

Abbreviations: CI, confidence interval; HR, hazard ratio; Ref., reference.

a. Unfavorable genotypes: ACACA rs7211875 (VV), rs11871275 (WW+WV).

b. Adjusted by gender,age,HBV,AFPlevel,stage,Differentiation,treatment.

c. Significant *P* values (<0.05).

**Table 4:** Cumulative Effect of Unfavorable Genotypes on Recurrence-free Survival of HCC Patients

Group (Number of Unfavorable Genotype)^a^	Recurrence/Total	HR(95% CI)^b^	*P*-value
Group 1(0)	4/11	Ref.	
Group 2(1)	214/372	**2.72(1.00–7.34)**	**0.05**
Group 3(2)	74/107	**3.43(1.25–9.43)**	**0.02**
*P* for trend			**<0.01**

Abbreviations: CI, confidence interval; HR, hazard ratio; Ref., reference.

a. Unfavorable genotypes: ACACA rs11871275 (WW+WV) and FASN rs4485435 (WV+VV).

b. Adjusted by gender,age,HBV,AFPlevel,stage,Differentiation,treatment.
