# Supplementary material for: Genetic variants in de novo lipogenic pathway genes predict the prognosis of surgically-treated hepatocellular carcinoma
Source: Sci Rep. 2015 Mar 26;5:9536. doi: 10.1038/srep09536 (PMC4379911; doi:10.1038/srep09536)
Supplement: Supplementary Information — supplementary figure 1 and table 1 [file srep09536-s1.pdf]

Genetic variants in de novo lipogenesis pathway genes predict the prognosis of surgical hepatocellular carcinoma

Hequn Jiang[1#], Jingyao Dai[2#], Xiaojun huang[3], Yibing Chen[3], Ping Qu[3], Jibin Li[3], Cheng Yi[1], Yefa Yang[4], Kejing Zhang[5*], Qichao Huang[3*]

[1]Cancer Center, West China Hospital, Sichuan University, Chengdu, Sichuan 610041, China.

[2]Department of Hepatobiliary Surgery, Xijing Hospital, Fourth Military Medical University, Xi'an, 710032, China.

[3]State Key Laboratory of Cancer Biology and Experimental Teaching Center of Basic Medicine, Fourth Military Medical University, Xi'an, 710032, China.

[4]Department of Interventional Radiology, Eastern Hepatobiliary Surgery Hospital, Second Military Medical University, Shanghai, 200438, China.

[5]College of Life Science, Northwest University, Xi'an, 710069, China.

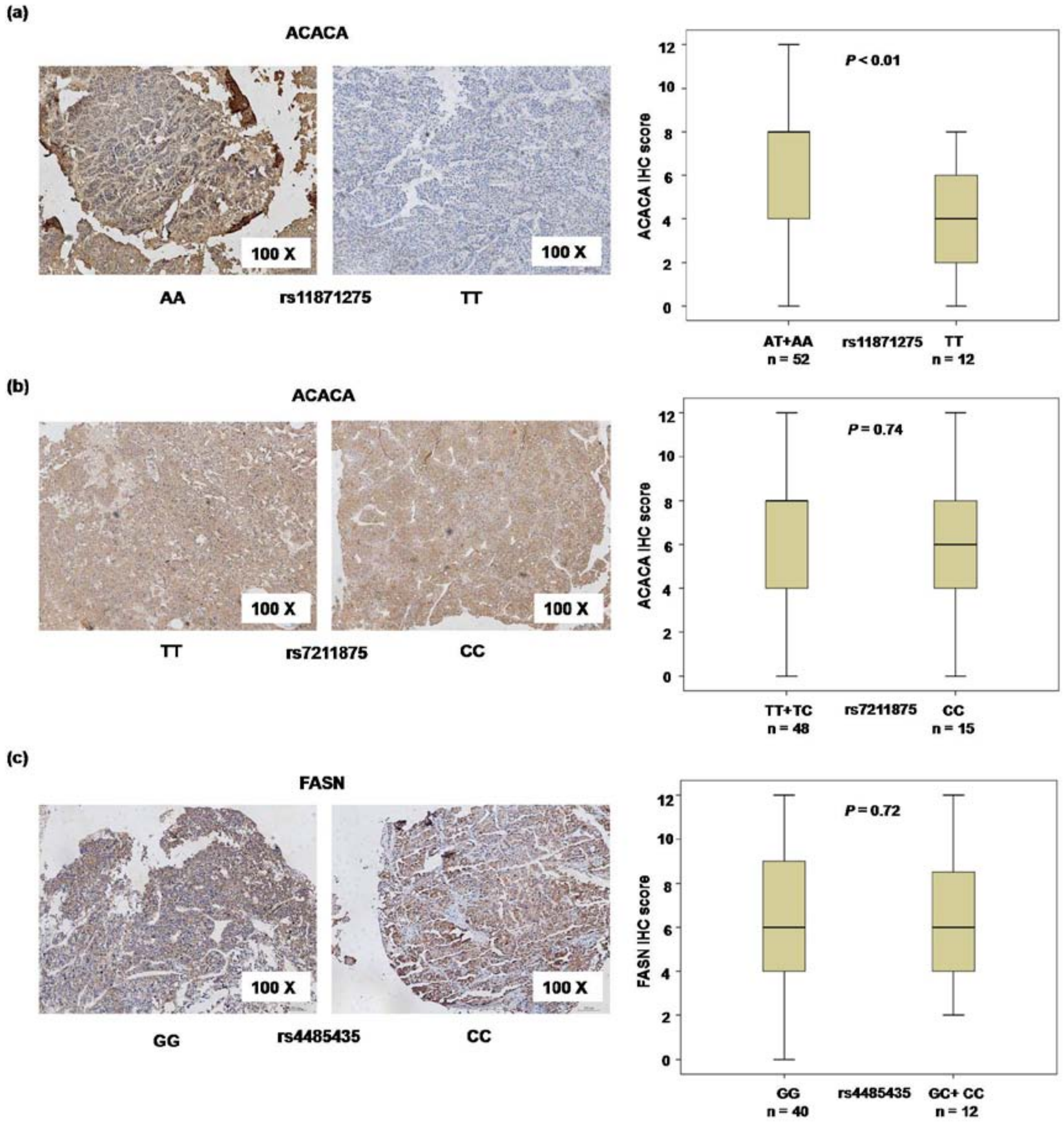

Supplementary figure 1.The relationship between SNP genotypes and the expression of ACACA and FASN in HCC tissue. (a) Immunohistochemical staining of ACACA and relationship between SNP rs11871275 genotypes and ACACA. (b) Immunohistochemical staining of ACACA and relationship between SNP rs7211875 genotypes and ACACA. (c) Immunohistochemical staining of FASN and relationship between SNP rs4485435 genotypes and FASN.

Supplementary Table 1. The basic information of targeted genes and their SNPs

Genes	SNPs	Allele	Functional analysis	Location /position (GRCh 37[illegible]
ACACA	rs1714987[a]	C/G	nsSNP/Splicing (ESE or ESS)	Intron (*ACACA*) /17: 357430[illegible]
	rs11871275	A/T	TFBS	Intron /17: 35769089
	rs7211875	T/C	Missense SNP (P[pro]>S[ser])	Exon /17: 35771468
ACLY	rs2304497	T/G	Missense SNP (E[Glu]>D[Asp])	Exon /17:40065774
	rs9912300	G/T	TFBS	Intron /17: 40025264
FASN	rs4246444	G/T	NA	Intron /17:80038952
	rs1140616	C/T	SynSNP	Exon /17:80039481
	rs4485435	G/C	SynSNP	Exon /17:80045086
	rs11653012	G/A	TFBS	3’ UTR /17: 80058857

Abbreviations: *ACACA*, Acetyl- CoA carboxylase- alpha; *ACLY*, ATP citrate lyase; *FASN*, Fatty acid synthetase; SNP, single nucleotide polymorphisms; nsSNP, non-synonymous SNP; TFBS, transcription factor binding site; SynSNP, synonymous SNP; NA, no information.

[a] GeneView data set indicated that rs1714987 was also a missense SNP in *C17orf78* gene.

[b] Genome Reference Consortium Human Build 37.5.
